# Supplementary material for: Characterization of the Fungitoxic Activity on Botrytis cinerea of N-phenyl-driman-9-carboxamides
Source: J Fungi (Basel). 2021 Oct 26;7(11):902. doi: 10.3390/jof7110902 (PMC8623464; doi:10.3390/jof7110902)

Compound 4: *2,5,5,8a-tetramethyl-N-phenyldecahydronaphthalene-1-carboxamide* or *N-phenyl-driman-9-carboxamide.*

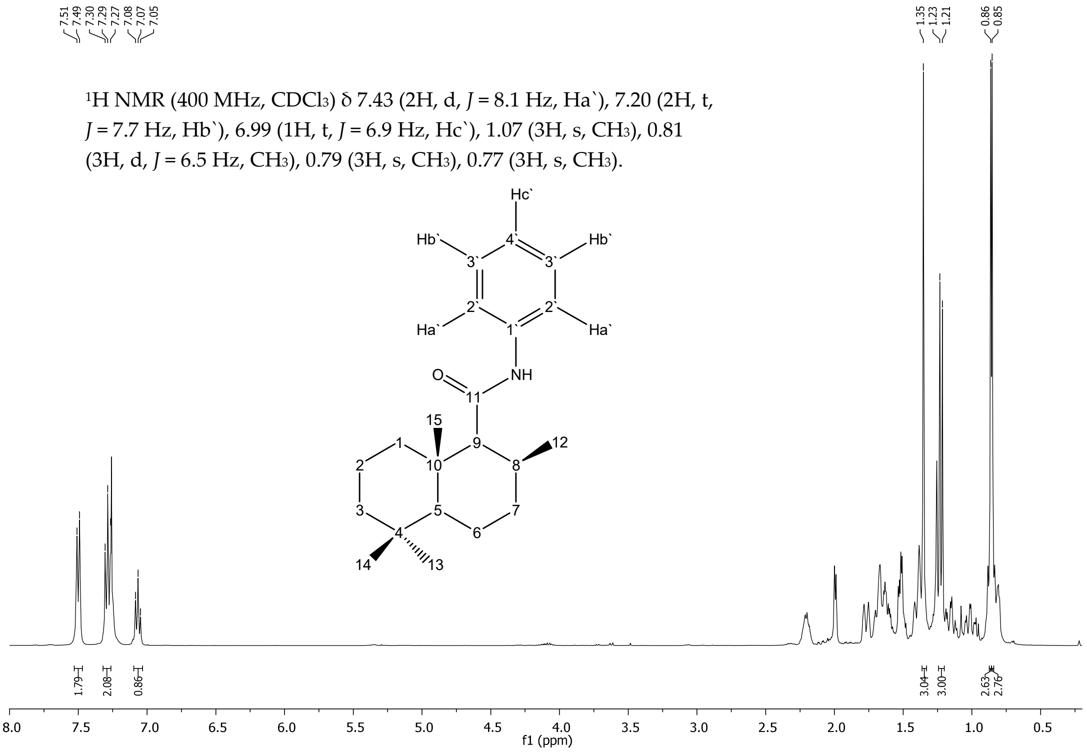

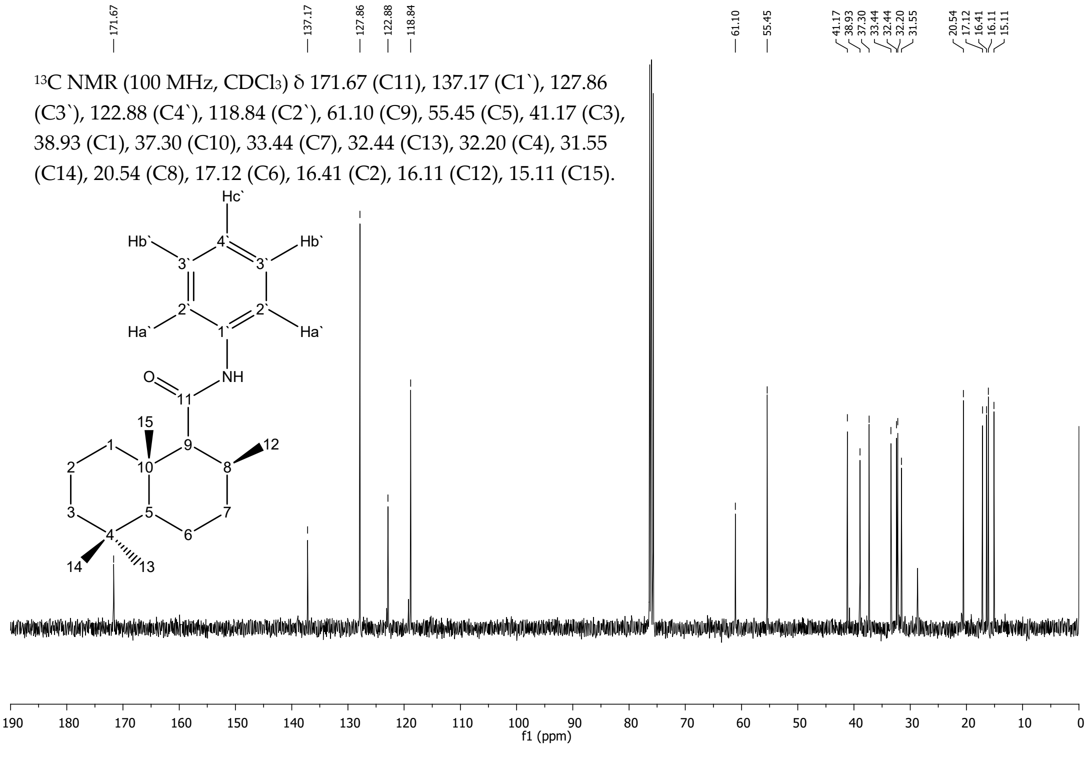

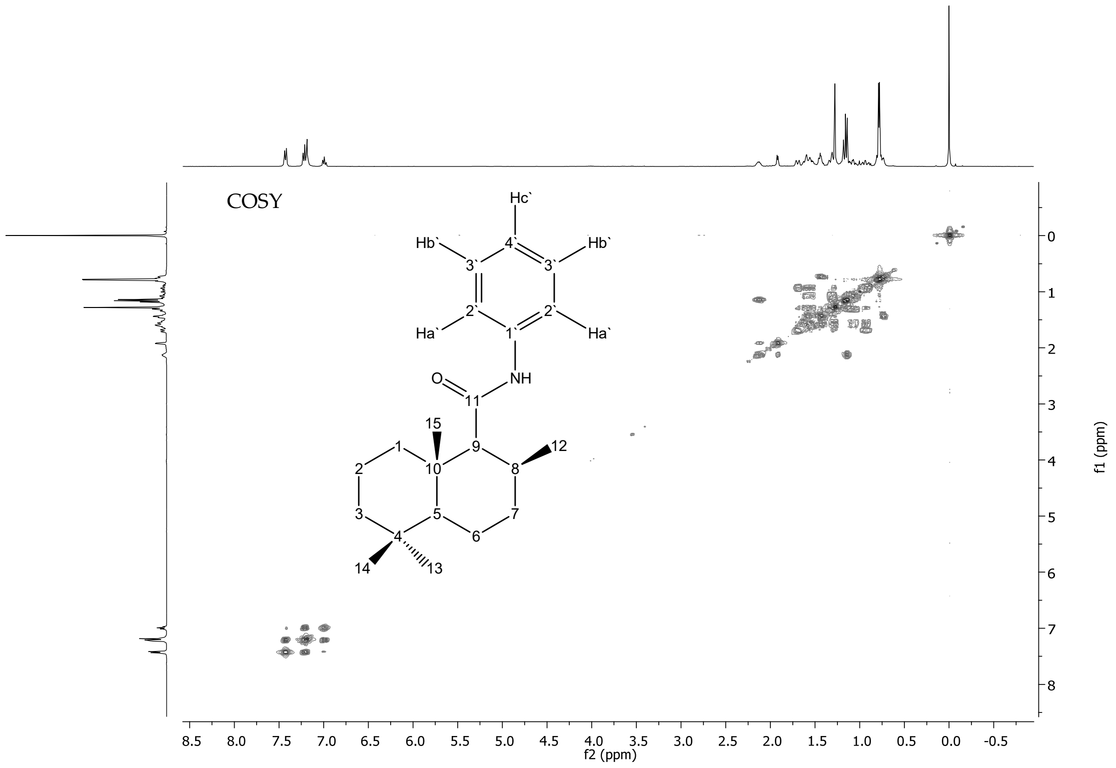
COSY
Hc`
Hb`
Hb`
Ha`
Ha`
NH
O
11
15
12
f2 (ppm)
f1 (ppm)

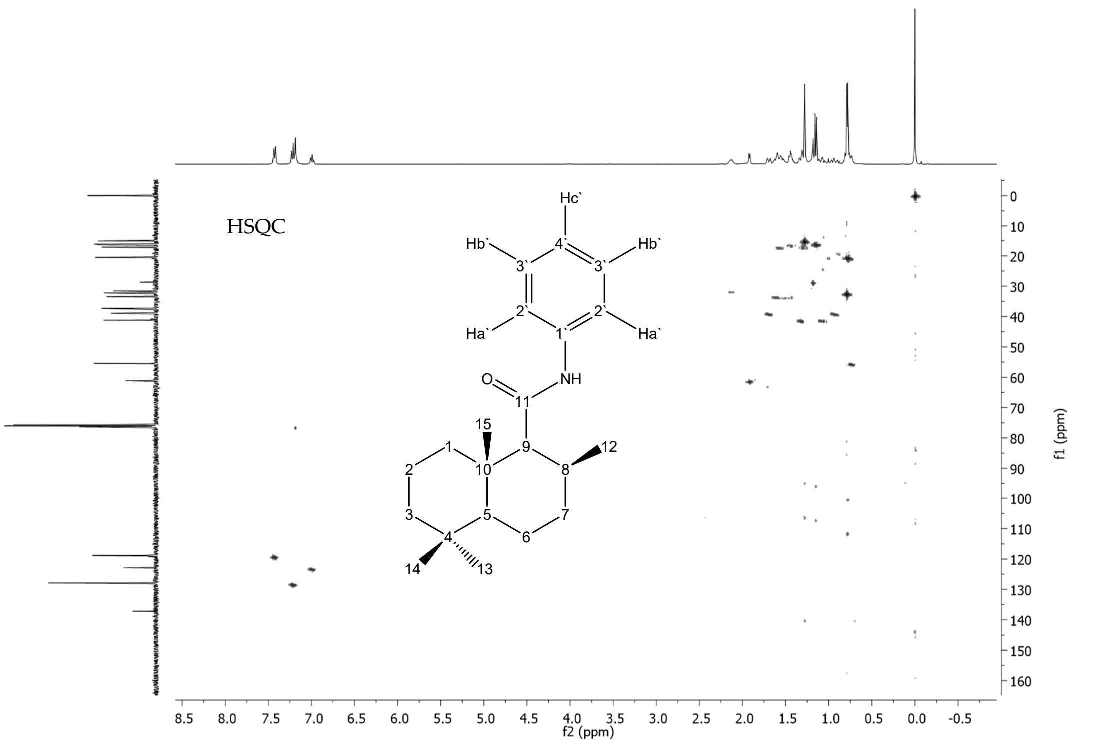
HSQC
Hc`
Hb`
Hb`
Ha`
Ha`
NH
O
11
15
12
f2 (ppm)
f1 (ppm)

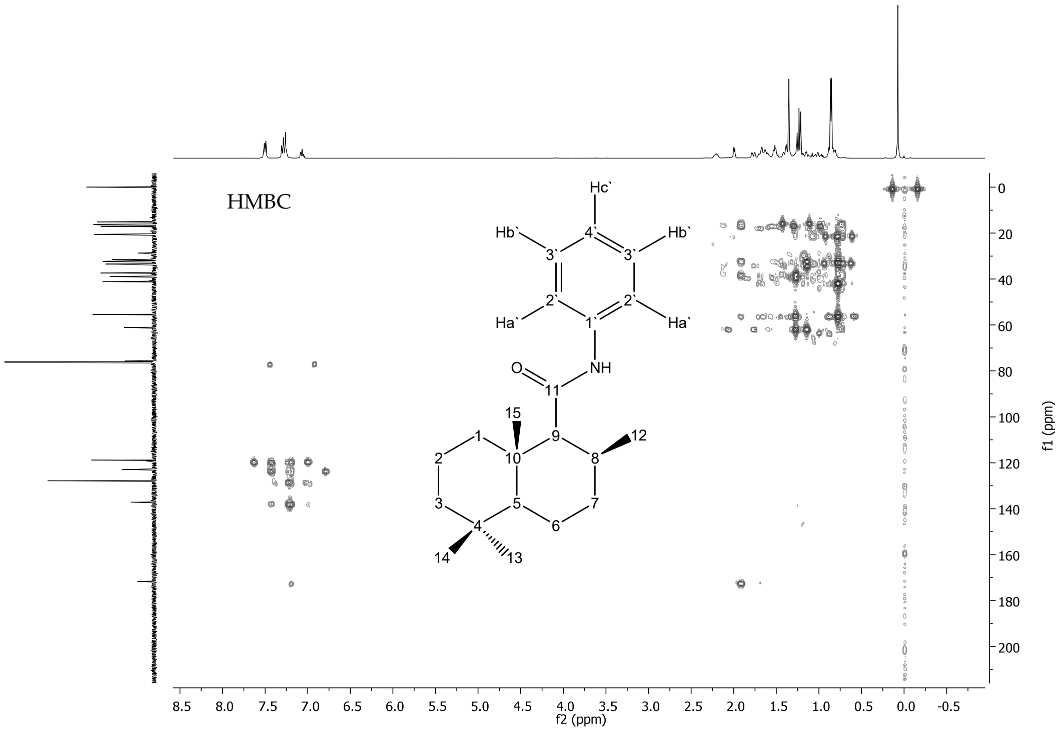
HMBC
Hc`
Hb`
4`
Hb`
3`
3`
2`
2`
Ha`
1`
Ha`
NH
O
11
15
9
1
12
2
10
8
3
5
7
4
6
14
13
f2 (ppm)
f1 (ppm)

Compound 5: *2,5,5,8a-tetramethyl-N-(3,4-dimethylphenyl)decahydronaphthalene-1-carboxamide* or *N-(3,4-dimethylphenyl)-driman-9-carboxamide.*

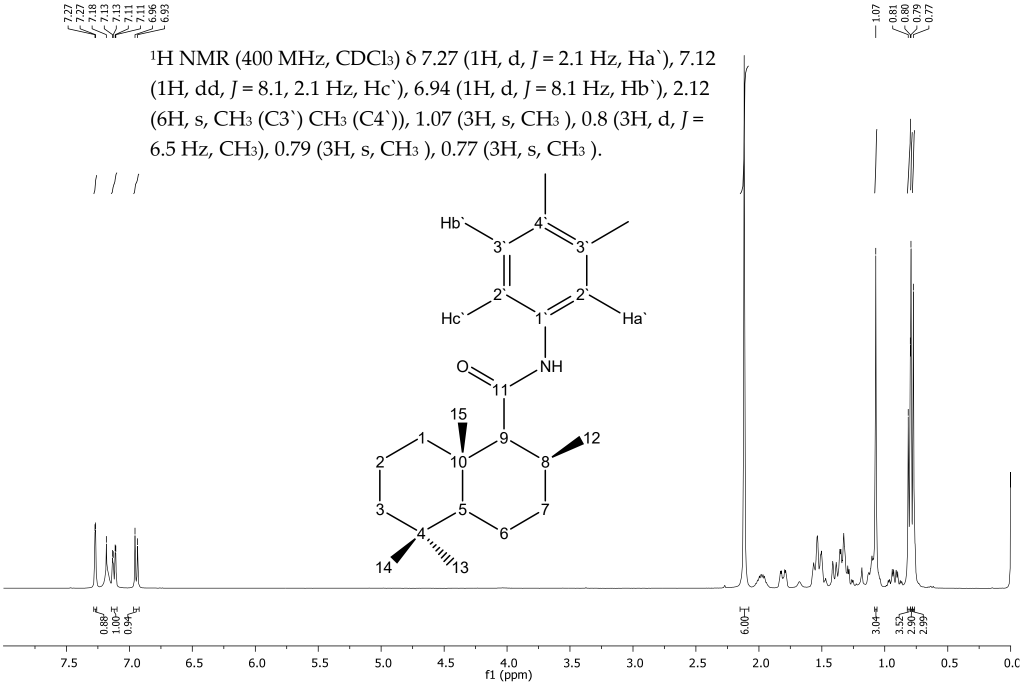

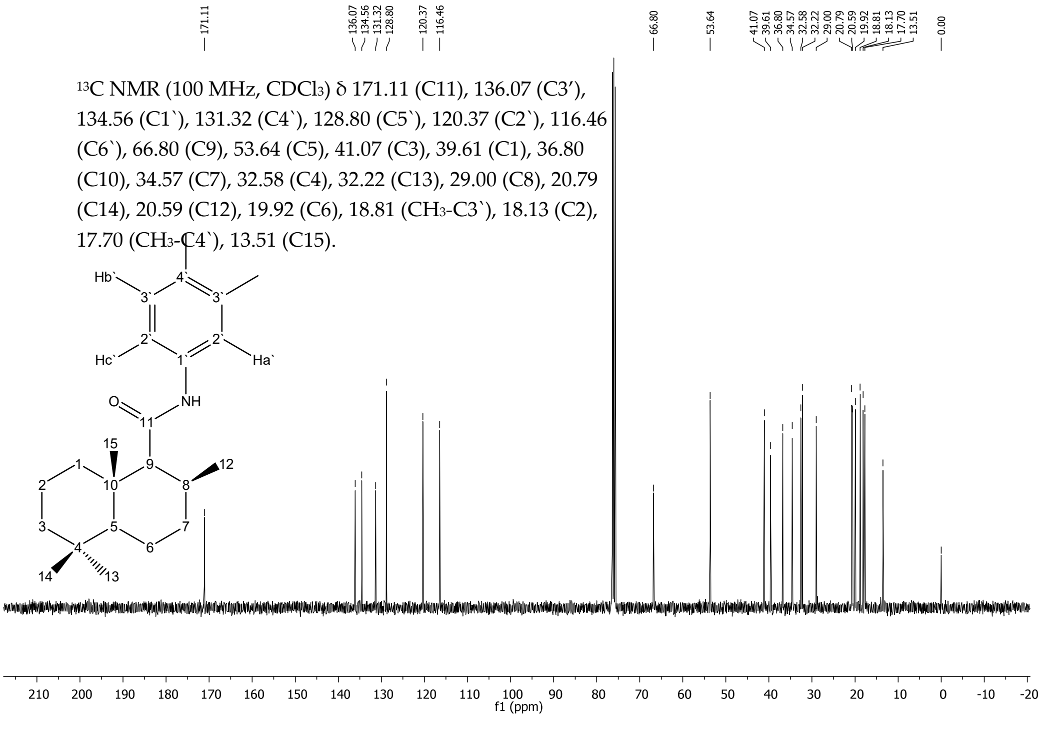

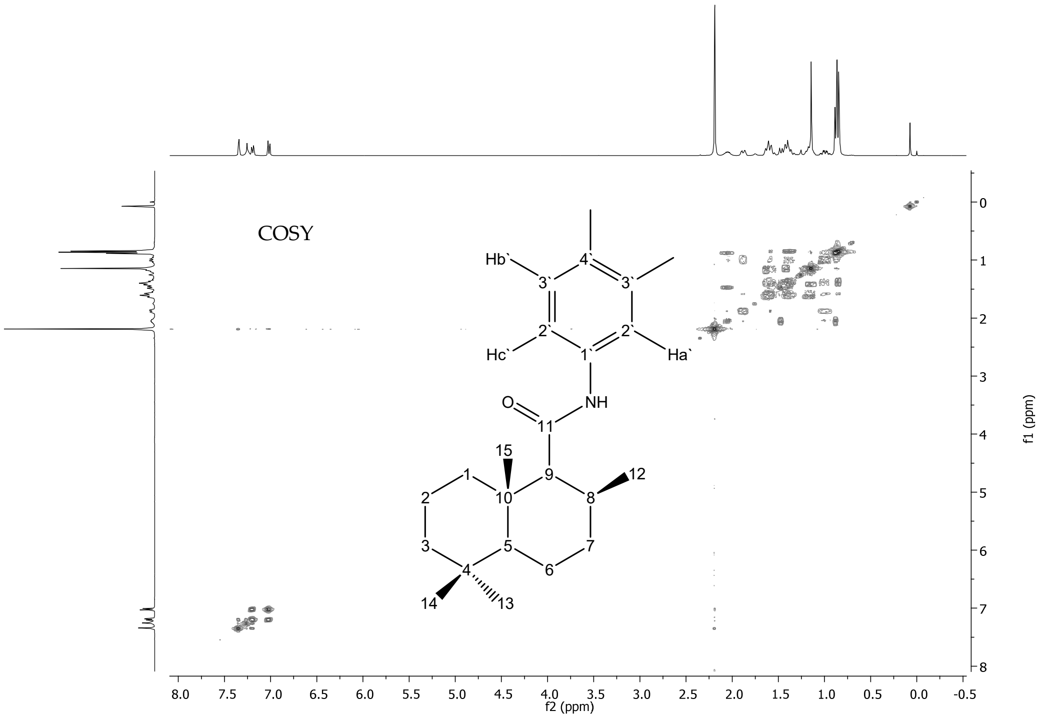
COSY
Hb`
4`
3`
3`
2`
2`
Hc`
1`
Ha`
O
NH
11
15
1
9
12
2
10
8
3
5
7
4
6
14
13
f2 (ppm)
f1 (ppm)

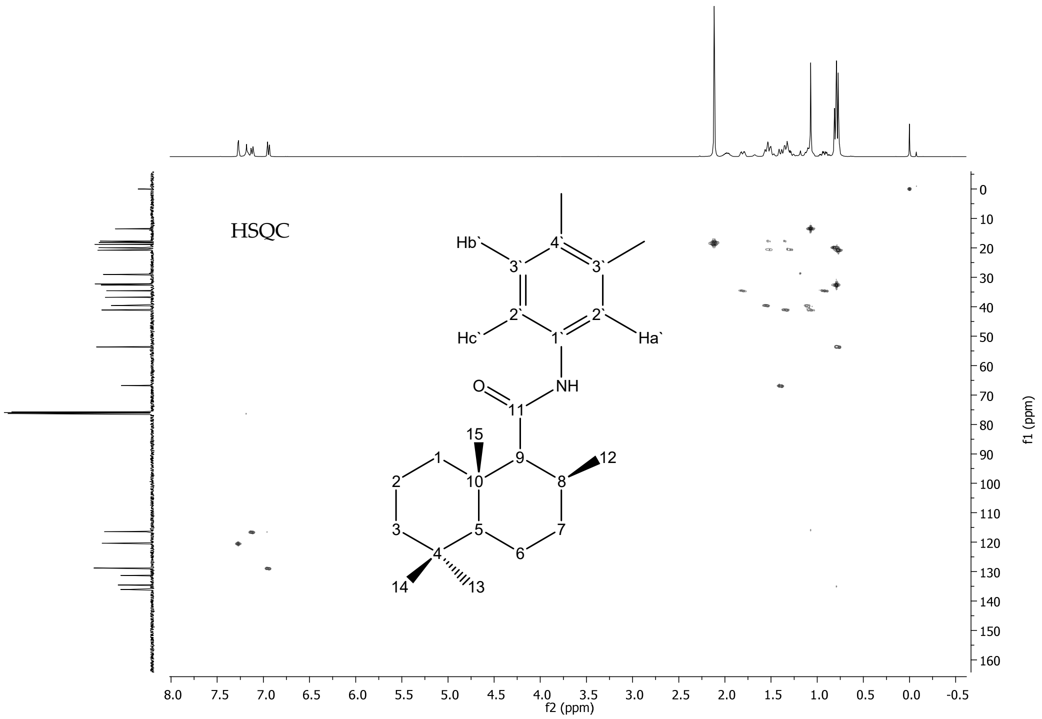
HSQC
Hb`
4`
3`
3`
2`
2`
Hc`
1`
Ha`
O
NH
11
15
1
9
12
2
10
8
3
5
7
4
6
14
13
f2 (ppm)
f1 (ppm)

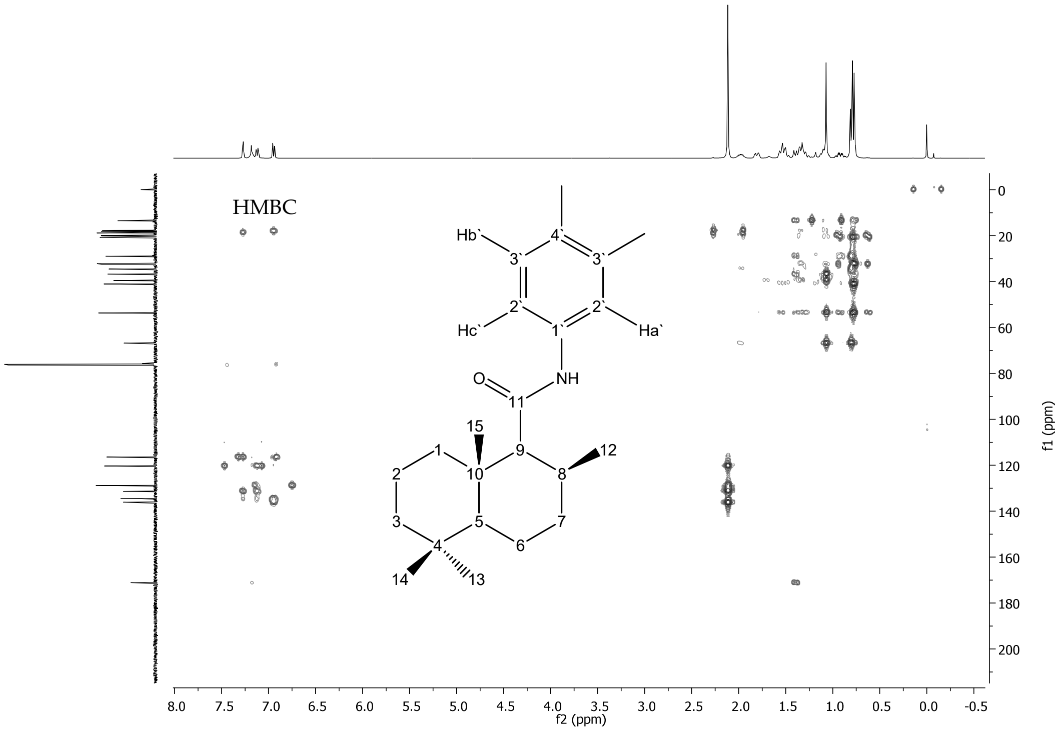
HMBC
Hb`
Hc`
Ha`
O
NH
f1 (ppm)
f2 (ppm)

Compound 6: *2,5,5,8a-tetramethyl-N-(3,4-dimethoxyphenyl)decahydronaphthalene-1-carboxamide* or *N-(3,4-dimethoxyphenyl)-driman-9-carboxamide.*

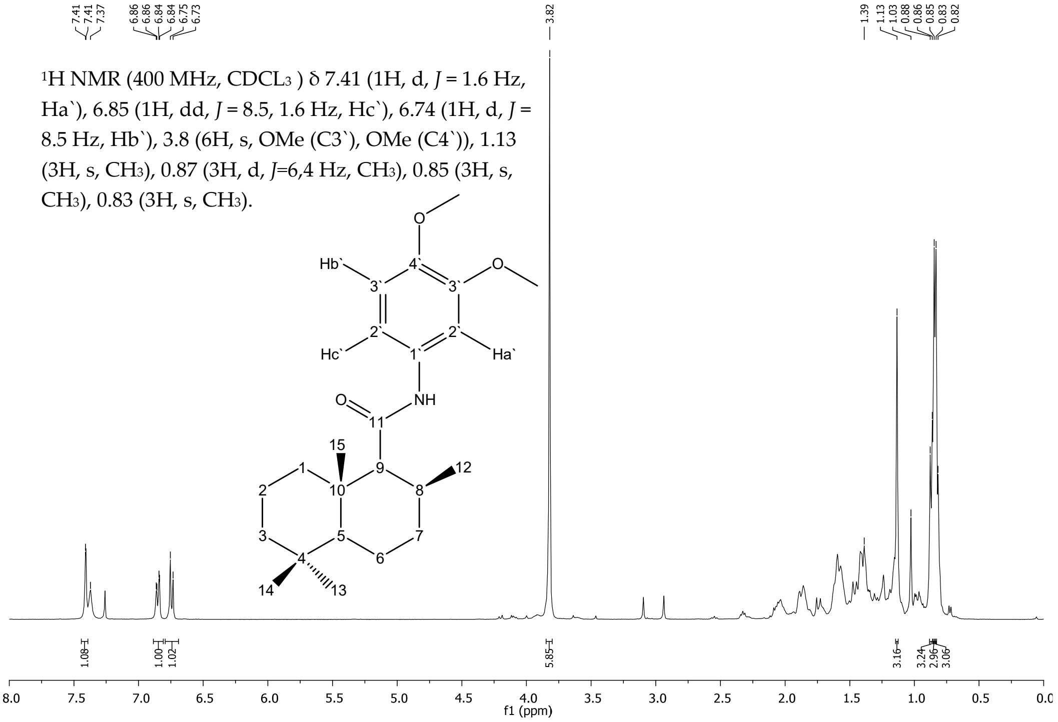

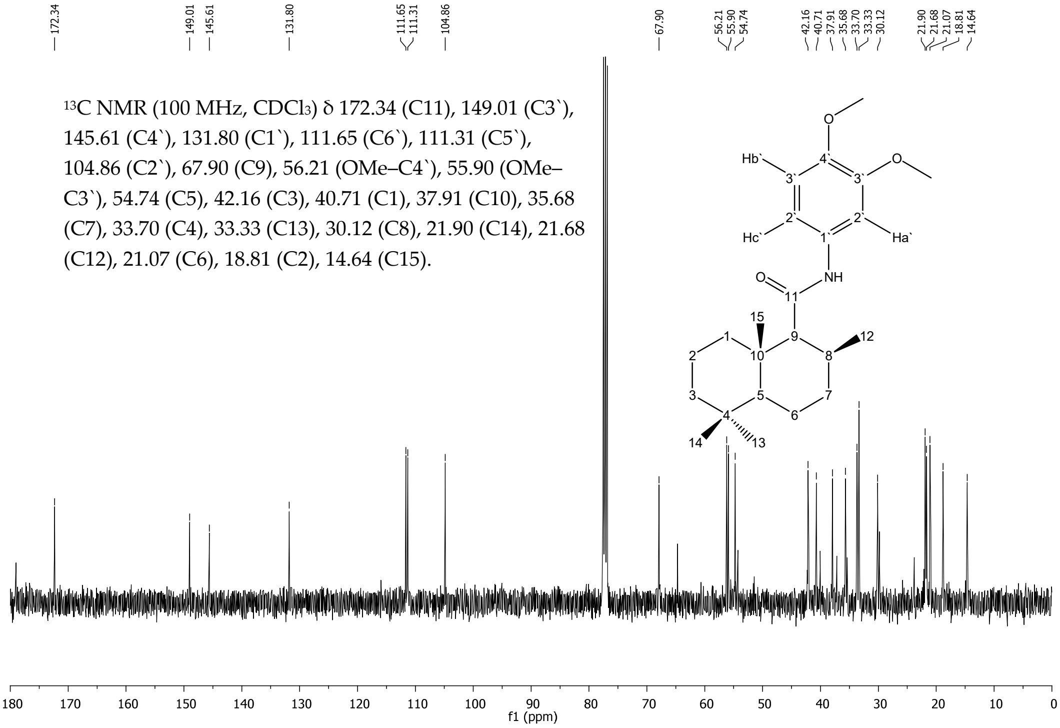

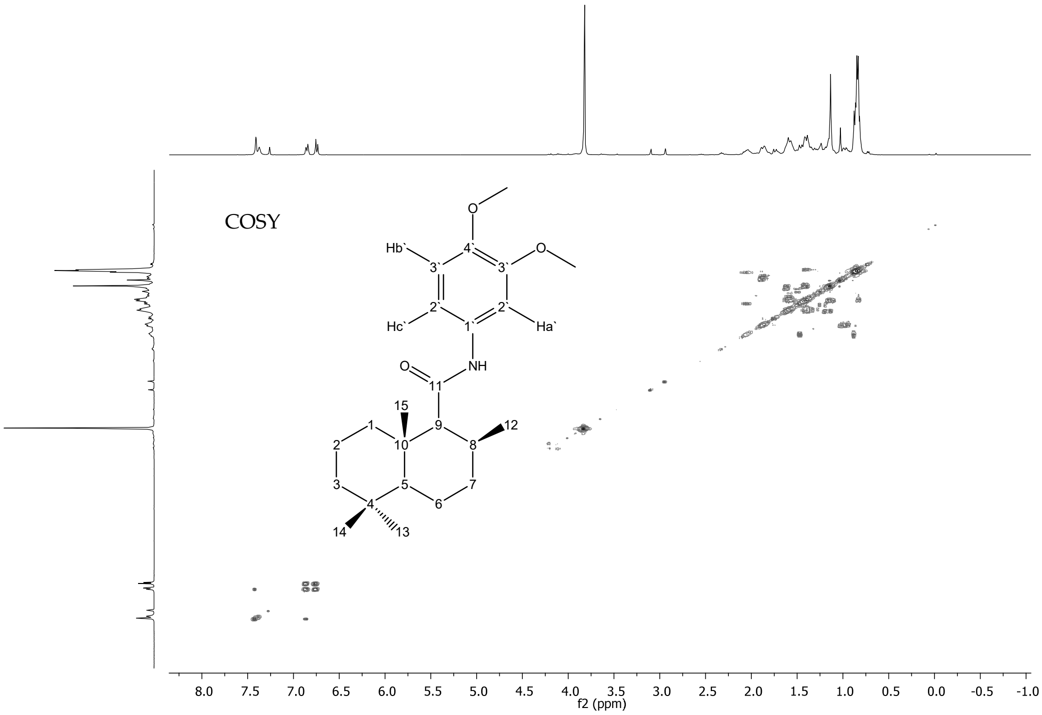
COSY
Hb`
Hc`
Ha`
NH
O
11
15
12
14
13
f2 (ppm)

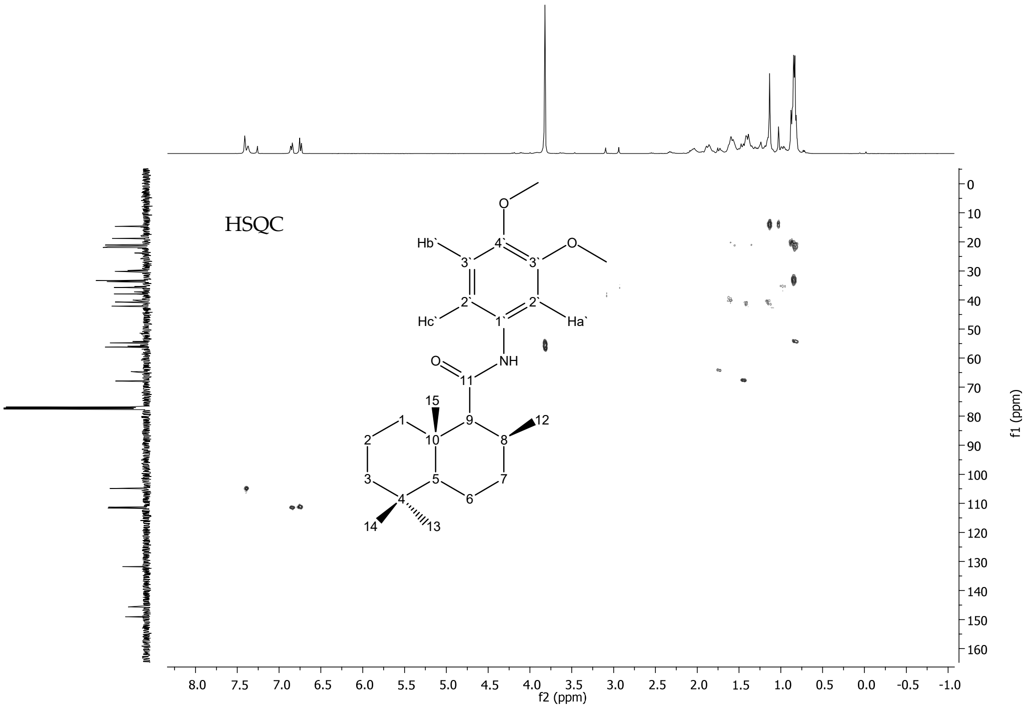
HSQC
Hb`
Hc`
Ha`
NH
O
11
15
12
14
13
f2 (ppm)
f1 (ppm)

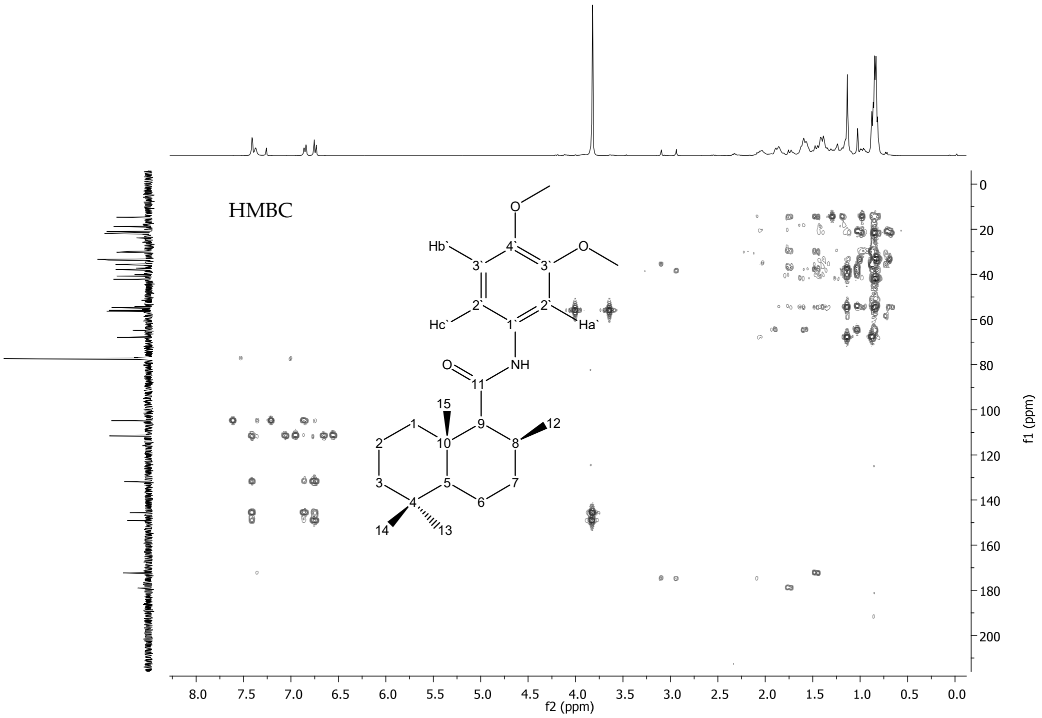
HMBC
Hb`
Hc`
Ha`
NH
O
f1 (ppm)
f2 (ppm)

Compound 7: *2,5,5,8a-tetramethyl-N-(3,4-dichlorophenyl)decahydronaphthalene-1-carboxamide or N-(3,4-dichlorophenyl)-driman-9-carboxamide.*

^{1}H NMR (400 MHz, $CDCl_3$) δ 7.75 (1H, s, Ha`), 7.31 (2H, d, *J* = 1.1 Hz, Hb` y Hc`), 1.12 (3H, s, CH_3), 0.86 (3H, s, CH_3), 0.85 (3H, s, CH_3), 0.84 (3H, s, CH_3).

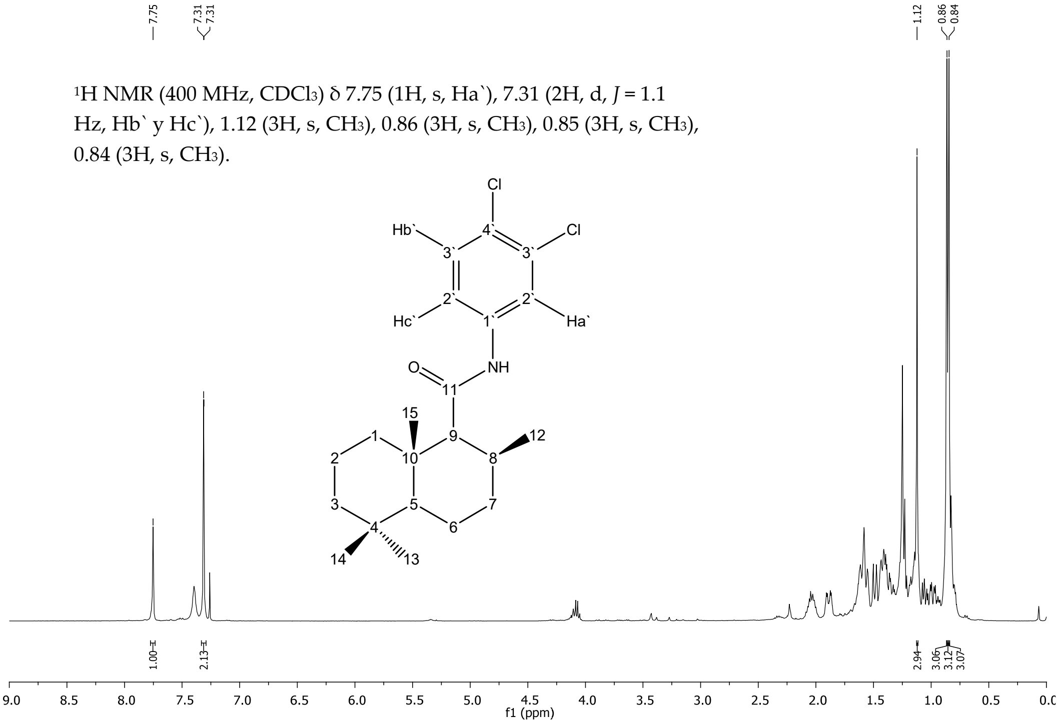

^{13}C NMR (101 MHz, $CDCl_3$) δ 172.73 (C11), 137.32 (C1`), 132.80 (C3`), 130.49 (C5`), 127.36 (C4`), 121.84 (C2`), 119.36 (C6`), 68.07 (C9), 54.80 (C5), 42.16 (C6), 40.88 (C1), 38.10 (C10), 35.64 (C7), 33.71 (C4), 33.39 (C13), 30.21 (C8), 21.92 (C14), 21.69 (C12), 21.03 (C6), 18.82 (C2), 14.65 (C15).

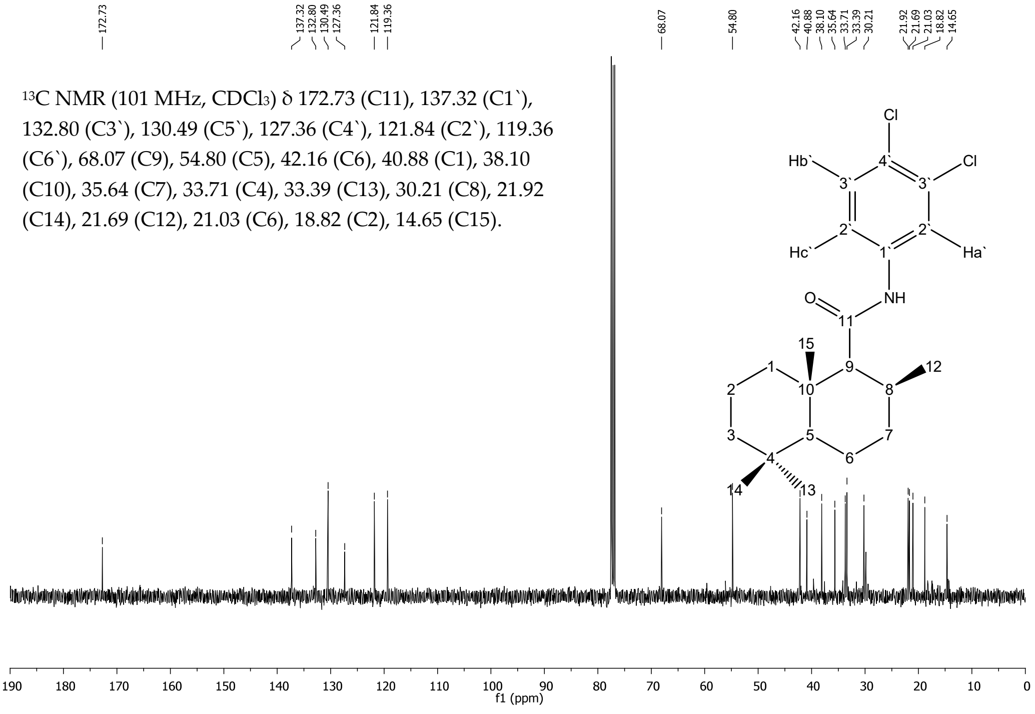

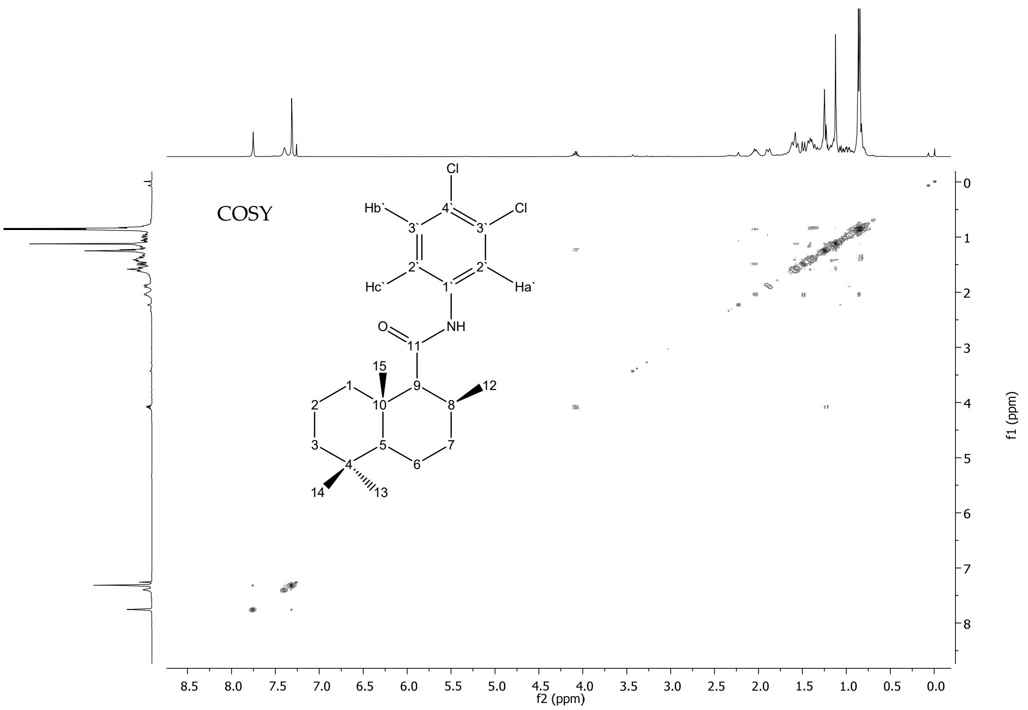
COSY
Cl
Hb`
Cl
Hc`
Ha`
O
NH
f1 (ppm)
f2 (ppm)

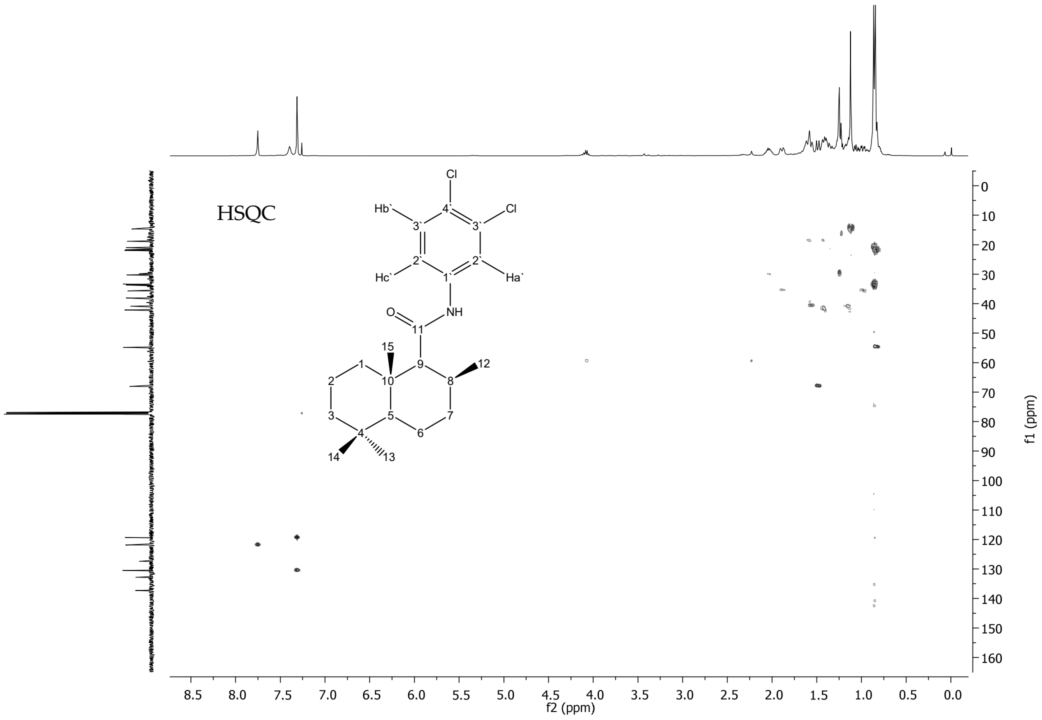
HSQC
Cl
Hb`
Cl
Hc`
Ha`
O
NH
f1 (ppm)
f2 (ppm)

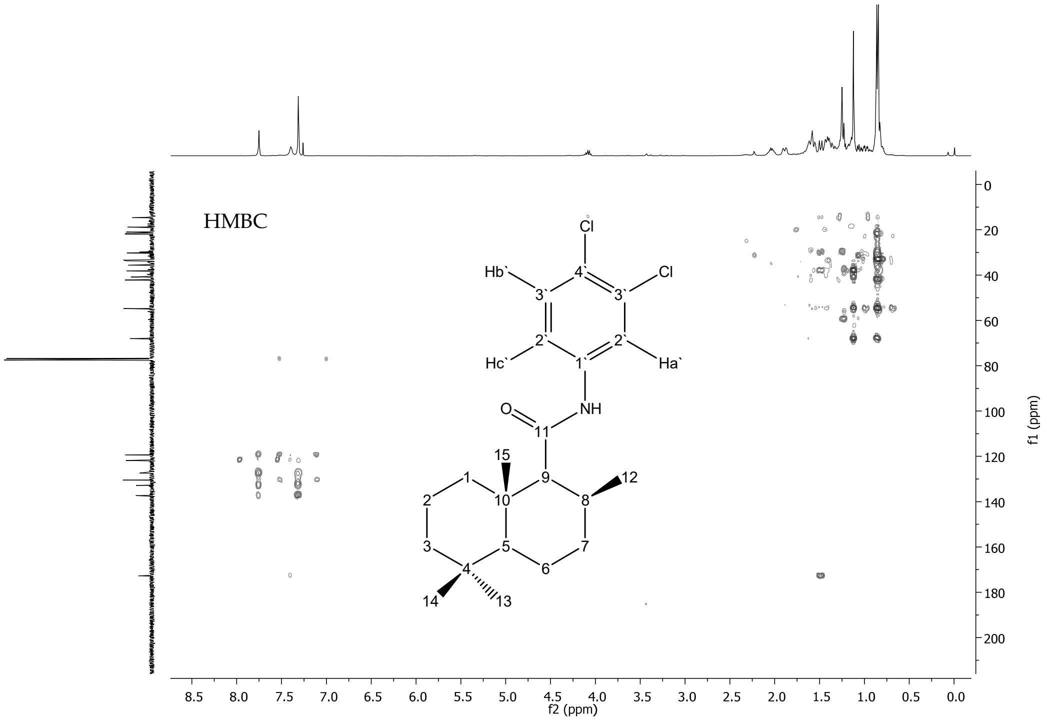
HMBC
Cl
Hb`
4`
3`
Cl
2`
1`
Hc`
Ha`
NH
O
11
15
1
9
8
12
2
10
3
5
7
4
6
14
13
f1 (ppm)
f2 (ppm)

Compound 8: *2,5,5,8a-tetramethyl-N-(3,5-dimethoxyphenyl)decahydronaphthalene-1-carboxamide or N-(3,5-dimethoxyphenyl)-driman-9-carboxamide.*

^{1}H NMR (400 MHz, $CDCl_3$) δ 6.78 (d, *J* = 1.7 Hz, 2H, Ha\`), 6.20 (t, *J* = 2.2 Hz, 1H, Hb\`), 3.76 (s, 6H, 2 OMe), 1.10 (d, *J* = 7.4 Hz, 3H, CH_3), 0.86 (s, 6H, $2CH_3$), 0.84 (s, 3H, CH_3).

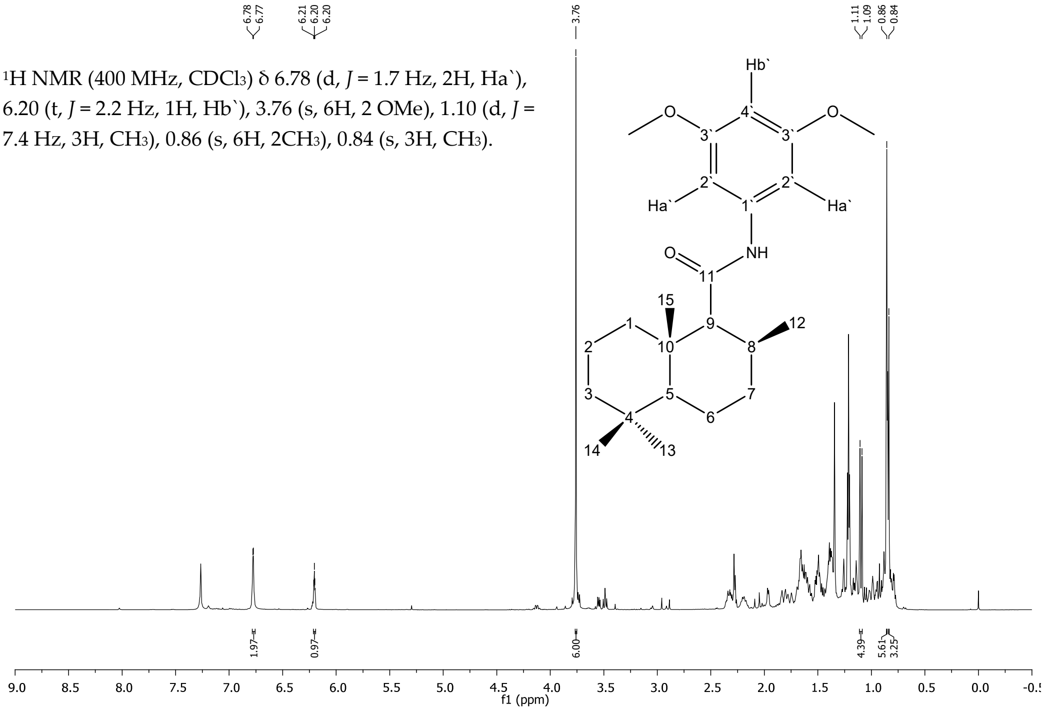

Compound 9: *2,5,5,8a-tetramethyl-N-(3,5-dichlorophenyl)decahydronaphthalene-1-carboxamide* or *N-(3,5-dichlorophenyl)-driman-9-carboxamide.*

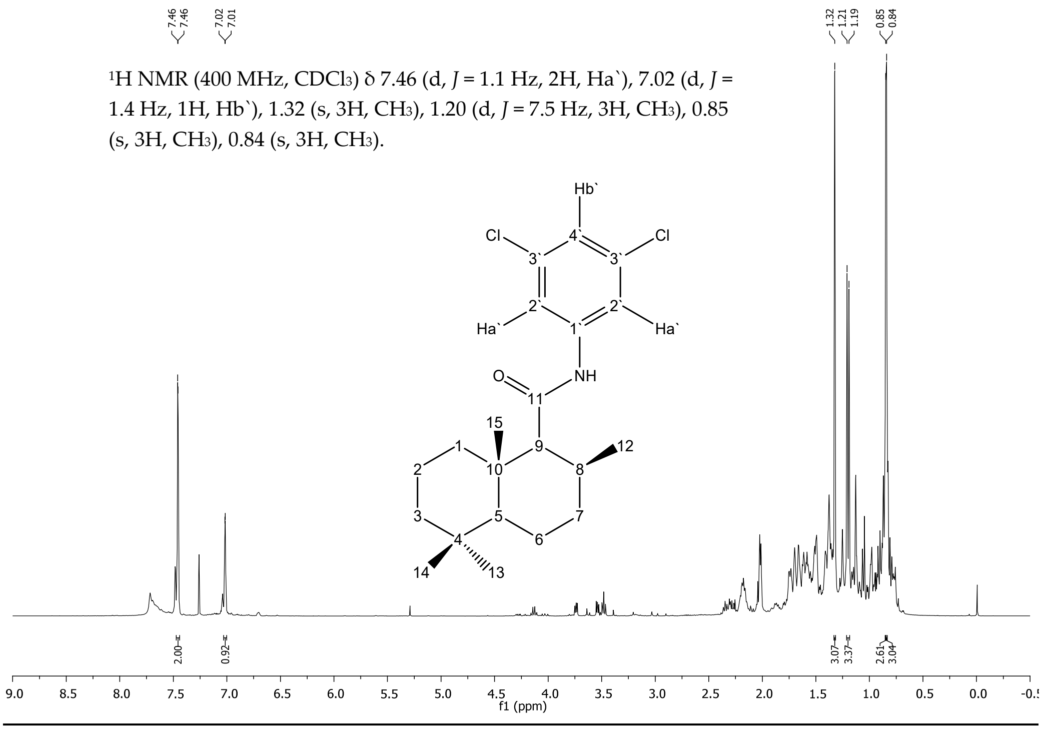

Compound 10: *2,5,5,8a-tetramethyl-N-(4-methylphenyl)decahydronaphthalene-1-carboxamide* or *N-(4-methylphenyl)-driman-9-carboxamide*

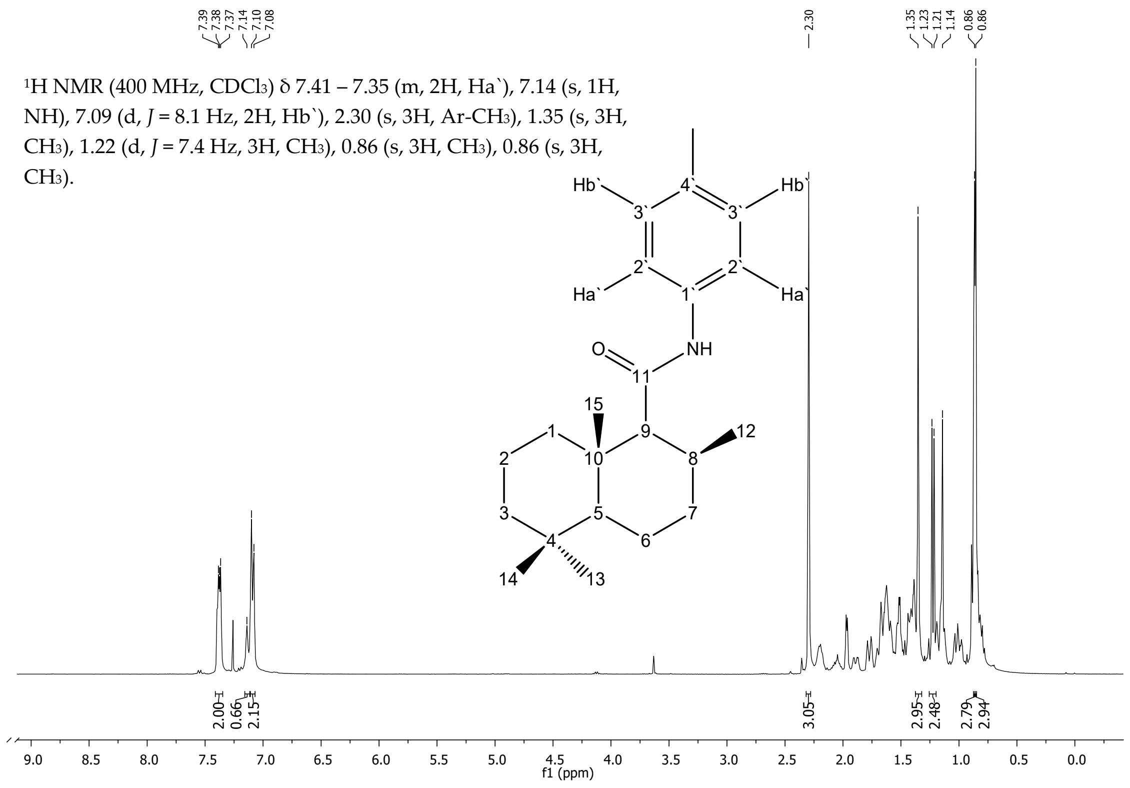

Compound 11: *2,5,5,8a-tetramethyl-N-(3-methylphenyl)decahydronaphthalene-1-carboxamide* or *N-(3-methylphenyl)-driman-9-carboxamide.*

^{1}H NMR (400 MHz, $CDCl_3$) δ 7.43 (d, J = 7.0 Hz, 1H, Hd`), 7.24 – 7.12 (m, 3H, Hc′, Ha`, NH), 6.89 (t, J = 5.9 Hz, 1H, Hb`), 2.32 (s, 3H, Ar–CH_3), 1.36 (s, 3H, CH_3), 1.23 (d, J = 7.4 Hz, 3H, CH_3), 0.87 (s, 3H, CH_3), 0.86 (s, 3H, CH_3).

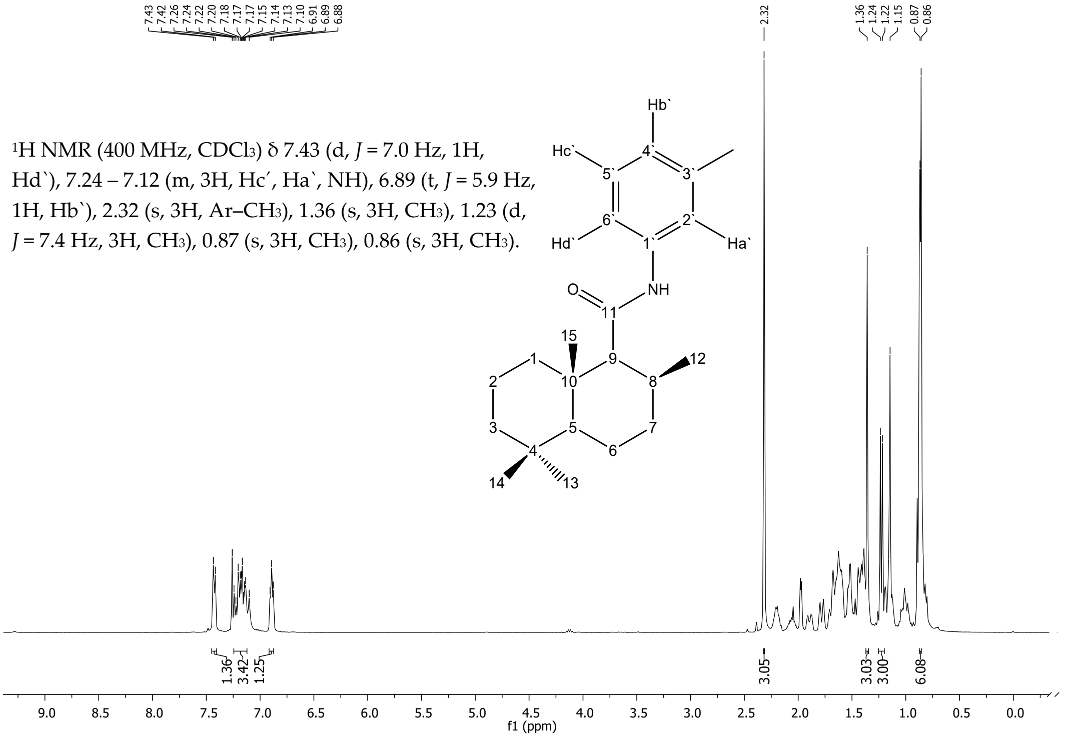

^{13}C NMR (101 MHz, $CDCl_3$) δ 172.62 (C11), 138.86 (C3`), 138.15 (C1`), 128.69 (C5`), 124.69 (C4`), 120.60 (2`), 116.94 (C6`), 68.15 (C10), 62.25 (C5), 56.53 (C3), 42.24 (C10), 40.02 (C1), 38.36 (C7), 34.52 (C8), 33.47(C13), 32.63(C14), 21.58 (Ar–CH_3), 18.18 (C6), 17.47 (C12), 17.14 (C2), 16.14 (C15).

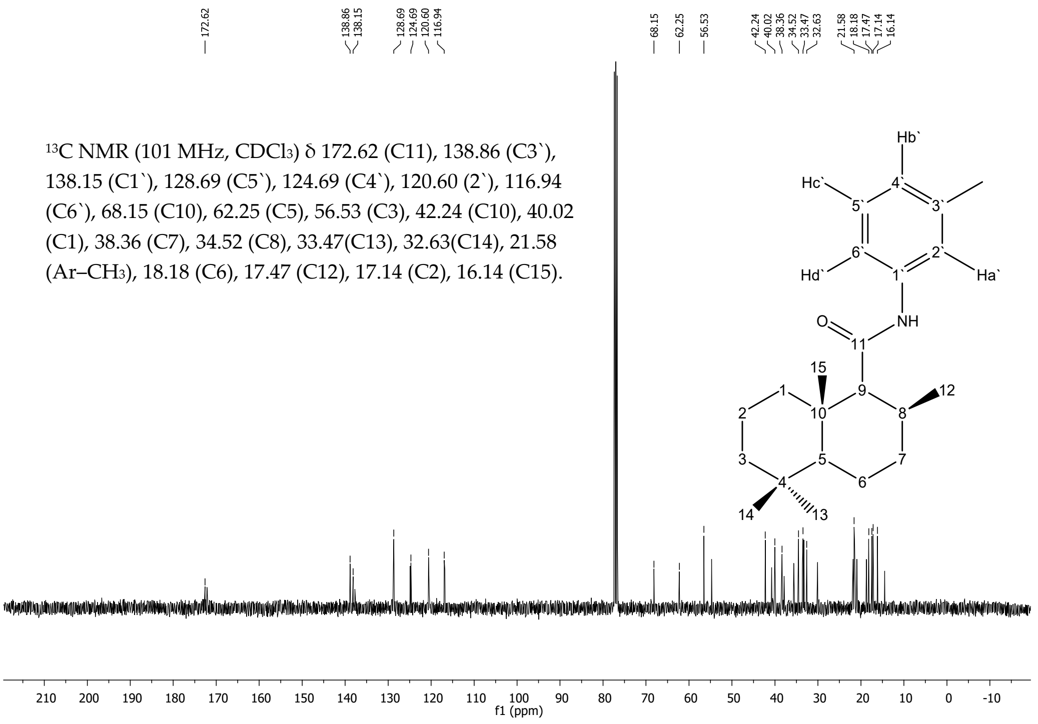

Compound 12: *2,5,5,8a-tetramethyl-N-(4-methoxyphenyl)decahydronaphthalene-1-carboxamide* or *N-(4-methoxyphenyl)-driman-9-carboxamide*

^{1}H NMR (400 MHz, $CDCl_3$) δ 7.32 (d, *J* = 8.3 Hz, 2H, Ha`), 6.74 (d, *J* = 8.4 Hz, 2H, Hb`), 3.69 (s, 3H, Ar–O–Me), 1.27 (s, 3H, CH_3), 1.15 (d, *J* = 7.4 Hz, 3H, CH_3), 0.78 (s, 6H, $2CH_3$)

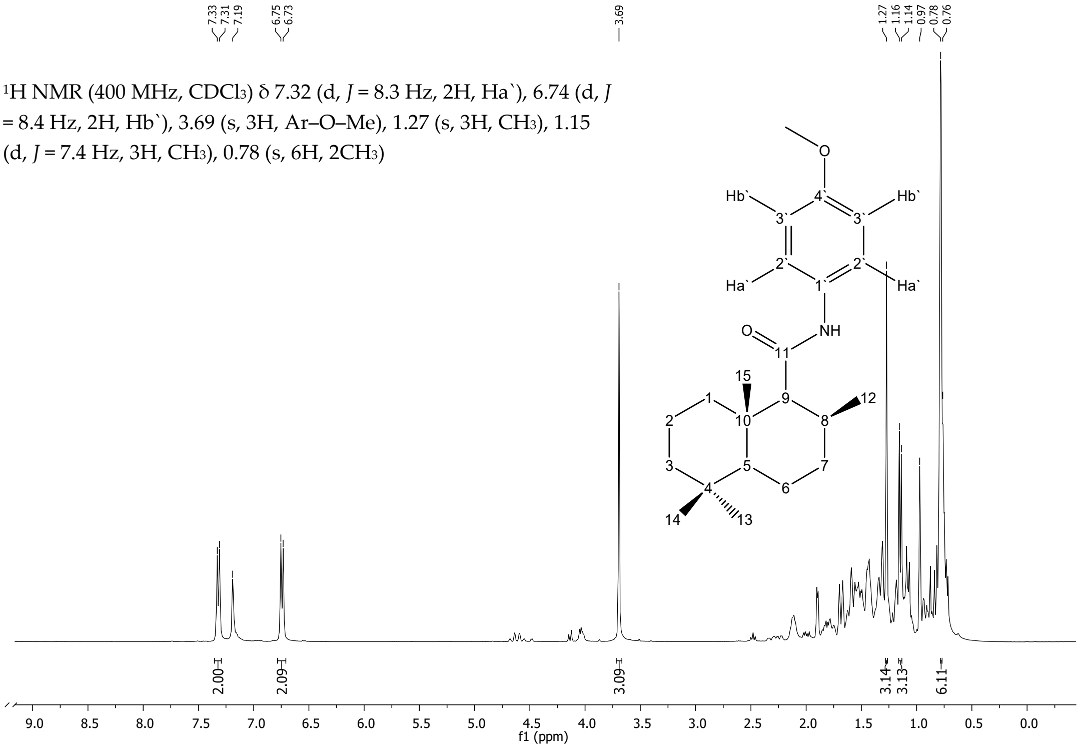

^{13}C NMR (101 MHz, $CDCl_3$) δ 172.57 (C11), 156.12 (C4`), 131.40 (C1`), 121.85 (C2`), 114.03 (C3`), 61.86 (C9), 56.50 (Ar–OMe), 55.49 (C5), 42.24 (C3), 39.97 (C10), 38.30 (C1), 34.52 (C7), 33.47 (C4), 33.23 (C8), 32.61 (C13), 21.58 (C14), 18.19 (C6), 17.47 (C12), 17.17 (C2), 16.16 (C15).

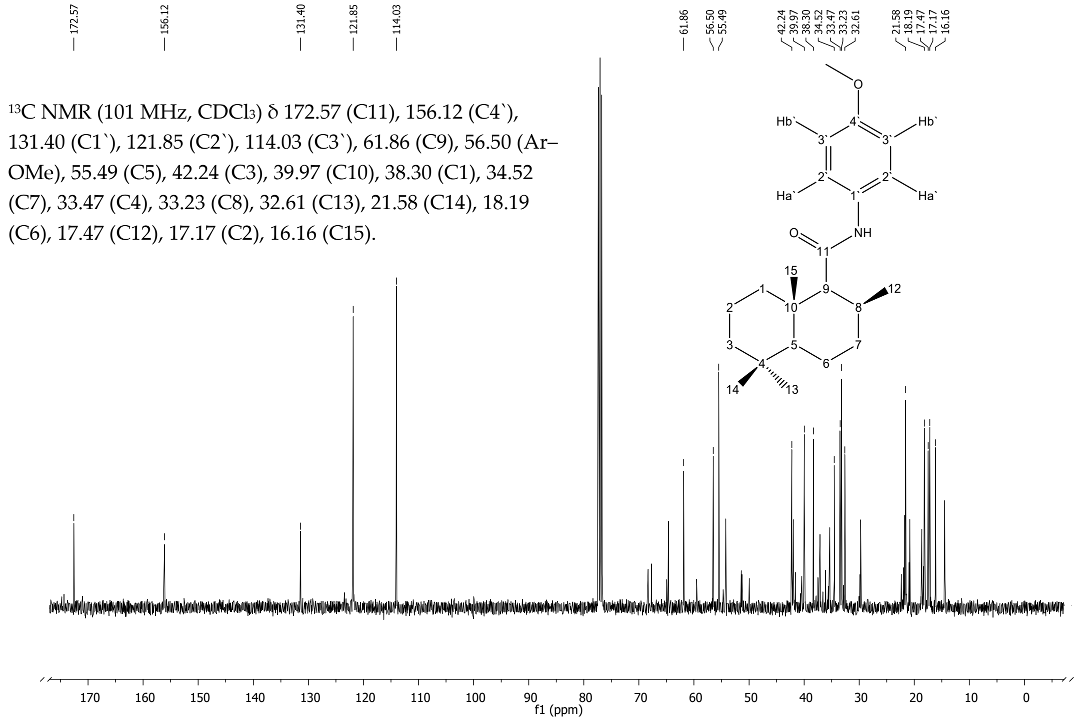

Compound 13: *2,5,5,8a-tetramethyl-N-(3-methoxyphenyl)decahydronaphthalene-1-carboxamide* or *N-(3-methoxyphenyl)-driman-9-carboxamide.*

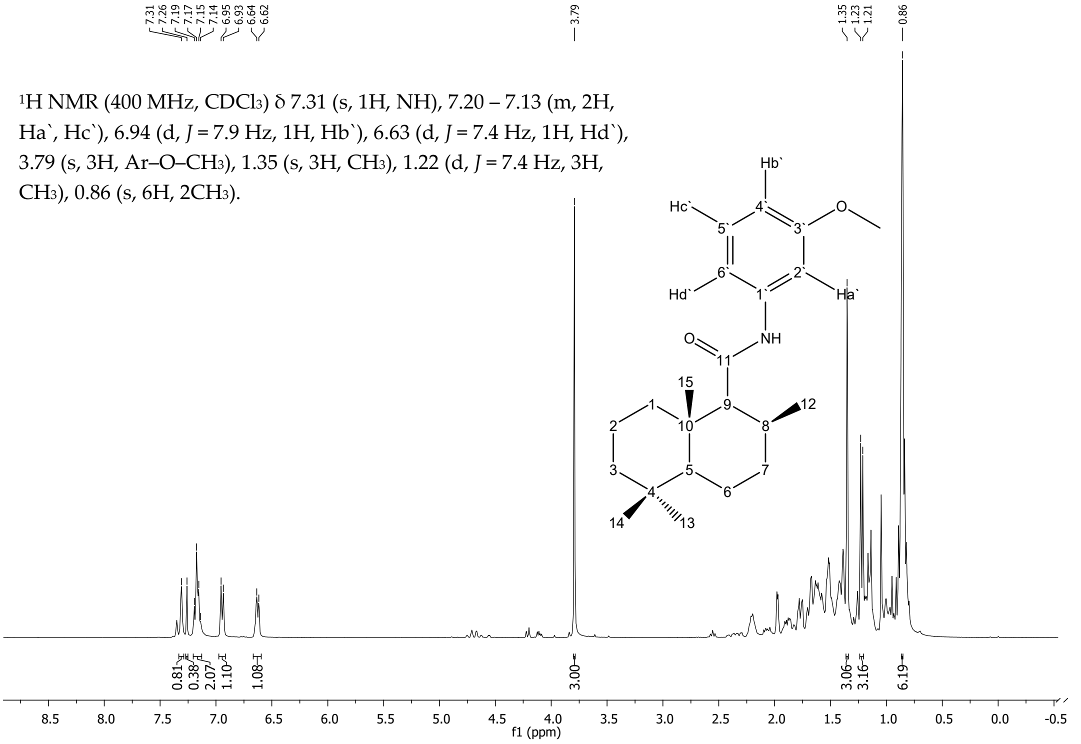

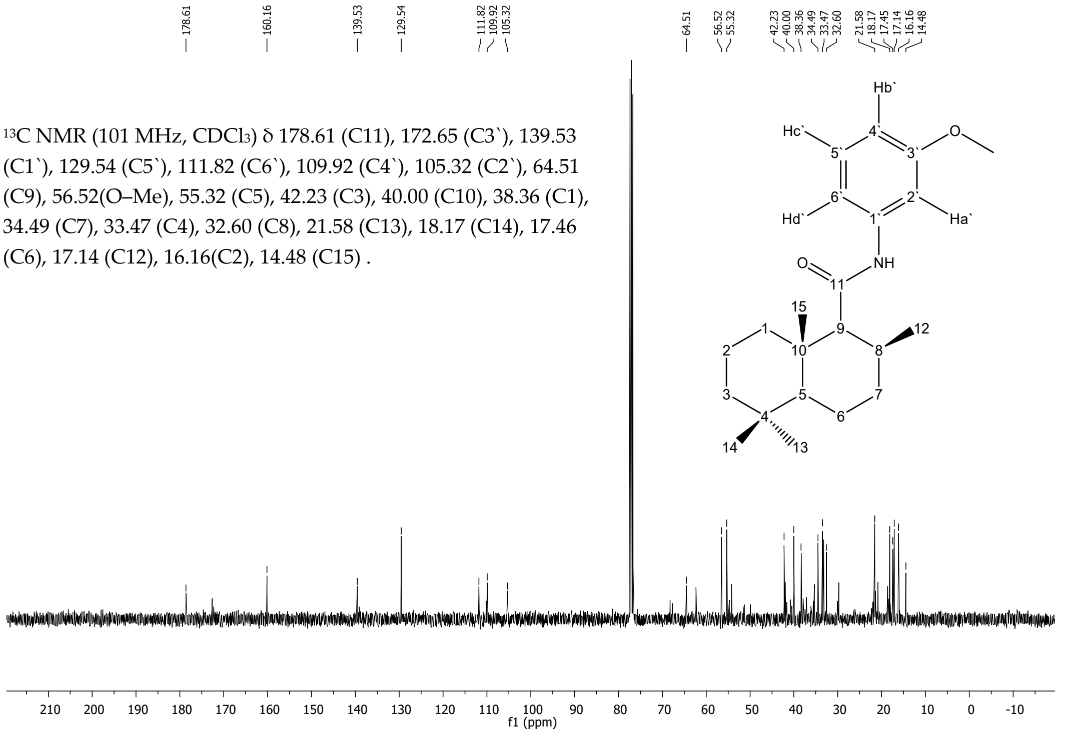

Compound 14: *2,5,5,8a-tetramethyl-N-(4-chlorophenyl)decahydronaphthalene-1-carboxamide or N-(4-chlorophenyl)-driman-9-carboxamide.*

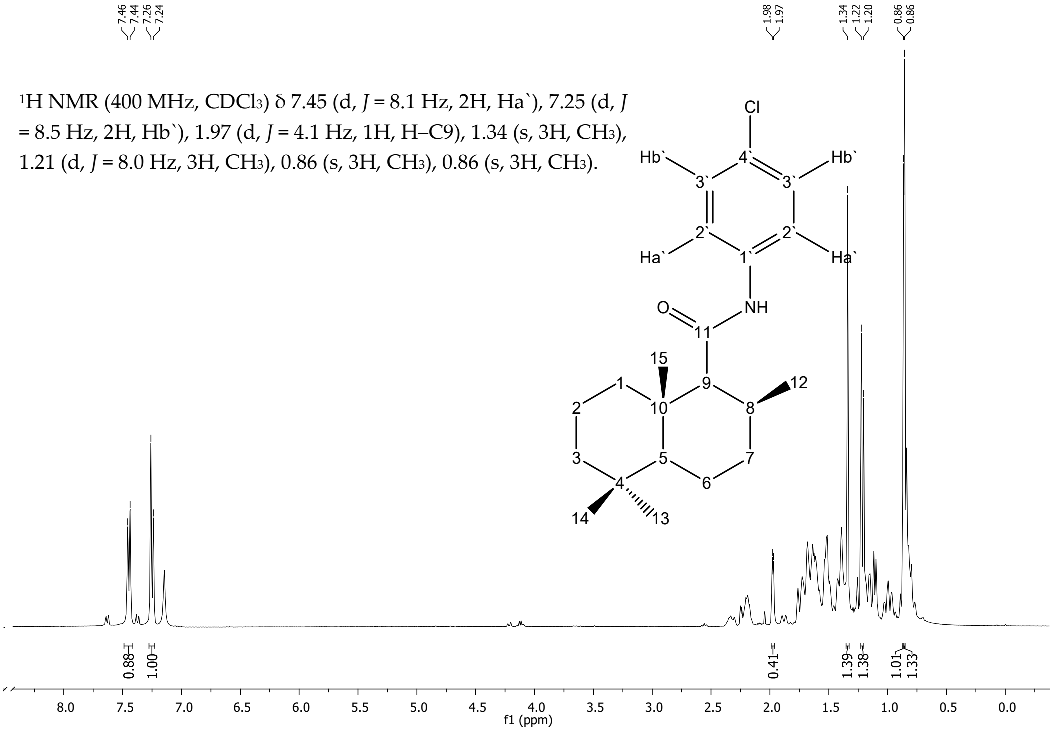

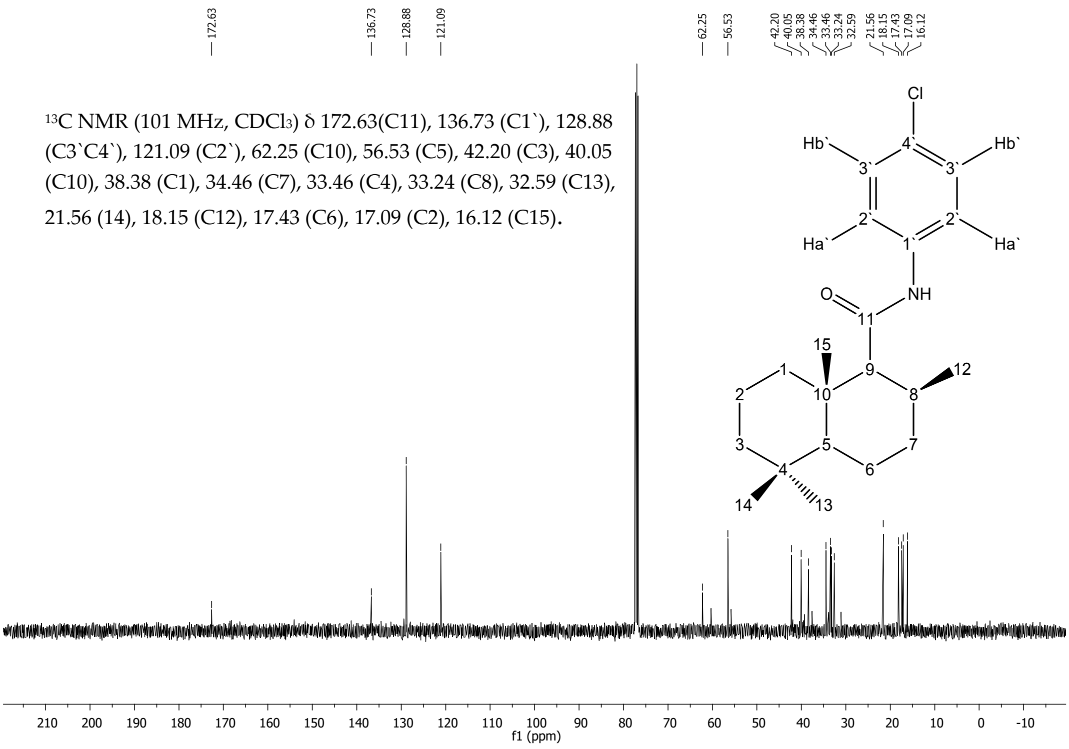

Compound 15: *2,5,5,8a-tetramethyl-N-(3-chlorophenyl)decahydronaphthalene-1-carboxamide or N-(3-chlorophenyl)-driman-9-carboxamide.*

^{1}H NMR (400 MHz, $CDCl_3$) δ 7.66 (s, 1H, NH), 7.29 (d, *J* = 8.3 Hz, 1H, Hd`), 7.20 (m, 2H, Ha`, Hc`), 7.04 (d, *J* = 7.9 Hz, 1H, Hb`), 1.98 (d, *J* = 4.2 Hz, 1H, H-C9), 1.34 (s, 3H, CH_3), 1.21 (d, *J* = 7.4 Hz, 3H, CH_3), 0.86 (s, 3H, CH_3), 0.85 (s, 3H, CH_3).

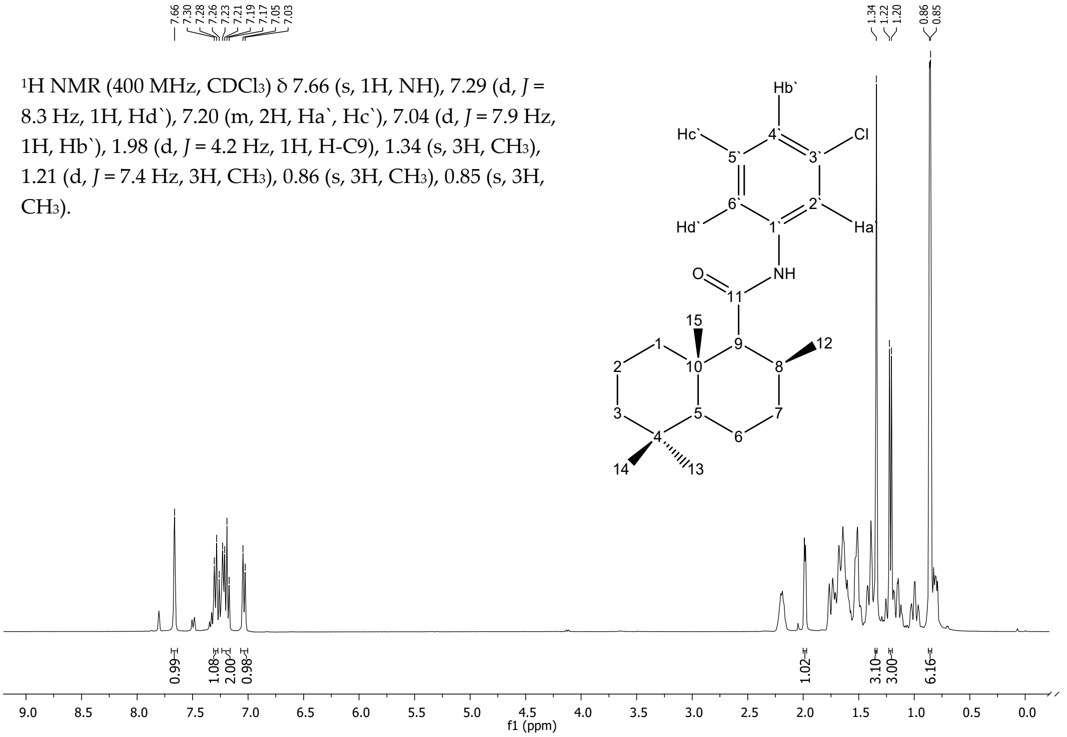

^{13}C NMR (101 MHz, $CDCl_3$) δ 172.78 (C11), 139.31 (C1`), 134.60 (C3`), 129.83 (C5`), 123.95 (C4`), 119.96 (C2`), 117.73 (C6`), 62.23 (C9), 56.49 (C5), 42.19 (C3), 40.03 (C10), 38.37 (C1), 34.44 (C7), 33.45 (C4), 33.24 (C8), 32.58 (C13), 21.56 (C14), 18.14 (C12), 17.42 (C7), 17.11 (C2), 16.13 (C15).

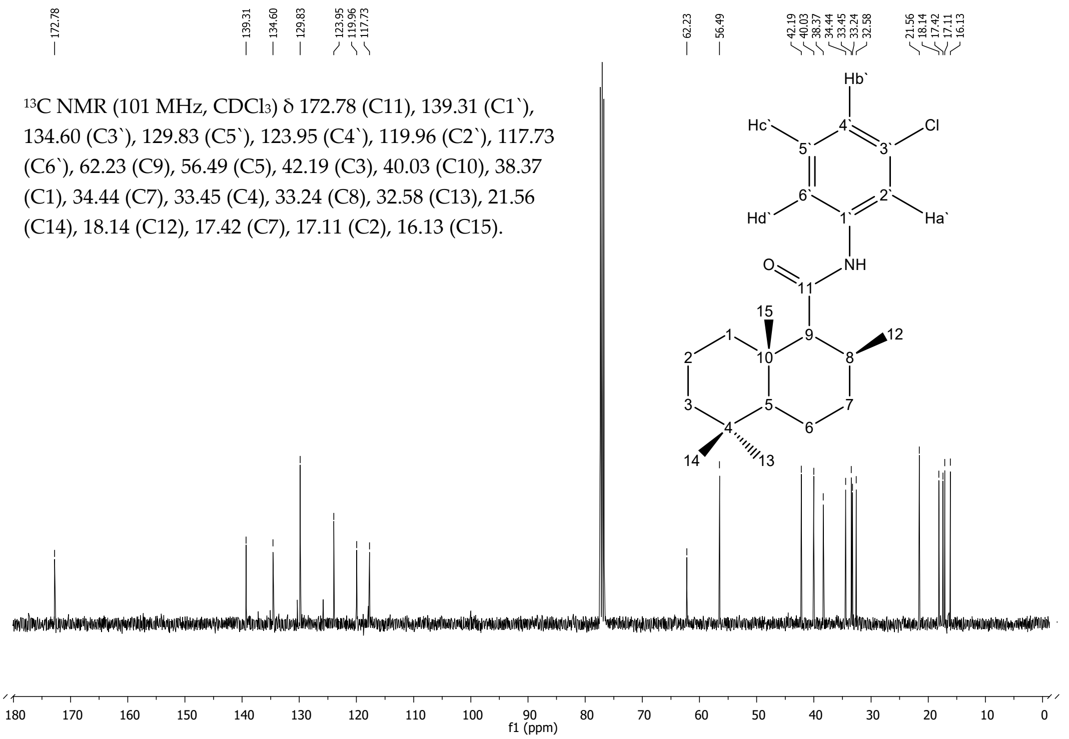

Supplement: Supplementary file 1 [file jof-07-00902-s001.zip › jof-1413592-supplementary.pdf]
